# Participatory epidemiology at the neotropics: study of diseases of backyard livestock and description of hunting patterns in Uaxactún, Maya Reserve Biosphere, Guatemala

**DOI:** 10.1186/s13104-016-2009-3

**Published:** 2016-04-07

**Authors:** Samuel Alberto Mérida Ruíz, Dennis Sigfried Guerra Centeno, Edgar Leonel Bailey Leonardo, Karl Rohn, Sarah Kösters, Lothar Kreienbrock

**Affiliations:** Department of Biometry, Epidemiology and Information Processing, University of Veterinary Medicine, Hannover, Bünteweg 2, 30559 Hannover, Germany; Institute of Animal Science and Ecohealth Investigation, Graduate School, Faculty of Veterinary Medicine, University of San Carlos, Guatemala, Ciudad Universitaria, zona 12, Guatemala City, Guatemala; Department of Zoo Sanitary Health, Ministry of Agriculture, Livestock and Food of Guatemala, 7a. avenida, 12-90 zona 13, Edificio Monja Blanca, Guatemala City, Guatemala

**Keywords:** Wildlife, Foodborne diseases, Risk factors, Food management, Cooking methods

## Abstract

**Background:**

The intention of the following study was to describe the interrelationship between villagers, domestic animals and wildlife at the Community Forestry Concession of Uaxactún, Guatemala by means of participatory epidemiological methods. The main focus was generating information regarding different livestock diseases considered important by villagers and their relevance, as well as obtaining knowledge concerning hunting activities and cooking methods to gain a better understanding of the interrelationship of people and animals and the diseases of their animals.

**Results:**

For poultry, an overall prevalence of 41 % of Newcastle disease was found by means of the ELISA test by antibody detection, chicken being the most affected species in the village. No samples were positive to avian influenza with the HI test. No virus was isolated by means of the tracheal or cloaca swabbing of ducks.

**For hunting:**

All species could be hunted by chance at any time of the year. There was a difference in species hunted between seasons, peccaries being more frequently hunted during the dry season and in contrast, deer and wild avian during the rainy season.

**For cooking:**

Villagers did not consume any raw meat. The cooking methods depended on the species. Stewing was the most favoured method for peccaries, wild birds, tepezcuintle and domestic poultry, whereas grilling was preferable for deer, roasting for armadillos and marinating for pork.

**Conclusion:**

According to the generated information, the most important domestic livestock species in the village are chickens and pigs, chickens being the most affected by diseases. No evident health problems on pigs were observed in this study. Hunting was shown as an activity enhanced by poverty and the lack of employment opportunities in the village and was mostly directed at larger species such as deer and peccaries. From the viewpoint of a transmission of zoonoses from animals to humans cooking methods mostly reflected a protective factor as no raw meat was eaten, stews and broths being the most common forms of cooking, involving an exposure of meat to high temperatures. Nonetheless, both agricultural and hunting activities represent a risk factor for the spread of diseases as hunters may act as mechanical vectors for different pathogens within domestic and wild animal populations.

## Background

In Guatemala, most veterinary services are found in and around the cities or close to the most inhabited places which are easily accessible. Conversely, for remote areas where environmental conditions are harsh and where access and infrastructure are poor, services are virtually absent. This increases the risk for disease misdiagnosis and dissemination as interaction between human and animal populations is a possible hazard for the spread of pathogenic agents, this being especially true for the strong interrelationship between wildlife and rural populations in developing countries [2].

Therefore, the intention of the following study was to describe and understand the diseases within the community of forestry concessions of Uaxactún, Maya Reserve Biosphere in Guatemala, as well as the behaviour of villagers towards wildlife.

In the study area, most of the families cultivate maize and other crops for domestic consumption and raise pigs and poultry in different amounts as sources of animal protein. They also collect and use material from wildlife. Hunting is a common activity happening on an opportunistic and irregular basis during the course of other agricultural activities, with only a small percentage of villagers relying on hunting for their subsistence [3, 4]. Subsistence hunting pressure is directed towards larger vertebrate species, and animals are generally harvested without regard to sex or age-class. According to Baur’s personal observations [4], the annual wildlife harvests represent a subsidy equivalent to more than 10 % of the local economy. Like many Maya Biosphere Reserve (forthwith: MBR) communities, Uaxactún is heavily dependent on natural resources to meet basic needs. However, reliance on bush meat differs between households as people have different consumption habits as well as different backyard animals. For villagers, subsistence hunting is an activity allowed and regulated by the National Council of Protected Areas (CONAP), following several requirements under the general law for hunting (Decree 36-2004 of the Republic of Guatemala). Not all hunters are legally registered within the CONAP database or are they willing to register within the CONAP.

One of the key constraints for veterinary surveillance and disease control is the lack of information on disease morbidity [26]. Governmental and non-governmental organisations regularly advise communities to conduct vaccination programmes for the prevention and control of animal diseases, without any real knowledge of the status of the diseases at those places. Since, in addition, the behaviour of the different diseases of livestock frequently changes depending on the season of the year and region of the country, generating knowledge for the creation of a baseline for further actions is required.

Nevertheless, gathering information within rural populations represents a challenge due to the difficulty in integrating those populations into structured information systems. Therefore, participatory epidemiology (PE) has been described as the use of participatory rural appraisal methods to improve the understanding of (animal) health issues, to collect, learn and to enable the local population to play a role in defining, analysing and solving their problems [9, 10, 15, 26]. PE is an emerging field in public health based on traditional epidemiological concepts on the one hand which allows for the exploration of interactions between the host, agent, and environment within a more social context on the other hand. The PE methodology is flexible, inexpensive, and employs a variety of techniques such as interviewing, mapping, and ranking to study disease patterns within a population and to identify pertinent information gaps [12].

The goal of the present study was to describe the veterinary and human health perceptions within the village under study by participatory approaches, as well as the various factors which could represent a risk for the health of villagers and their domestic livestock. Aspects concerning hunting and meat cooking methods were explored as well. Results concerning interviews, personal observation and comments of villagers are summarised.

## Methods

### Study area

The study was performed in Uaxactún [subtropical moist forest at 17°23′40.41′′ N, 89°37′58.29′′ W 168masl (UTM 16q 220082.10 mO, 1925018.32 mN)], located in the north of Guatemala having 688 inhabitants in 2000 and about 1000 in 2008. Uaxactún is one of the largest and oldest traditional forest settlements within the Maya Biosphere Reserve itself. The study took place from the months of August 2013 to May 2014.

### Participatory epidemiology

The interrelationship between Wildlife—Domestic animals—human was determined through participatory methods [7, 10, 15]. The target population was defined as the whole community, of which people who accepted to participate, with different degrees of engagement, were enrolled for the survey. Sampling strategy was a non-probabilistic approach by convenience [11]; all houses were subject to be included, nevertheless, not all people were willing to collaborate. Among the study participants 26 housewives and 10 hunters engaged actively in the investigation. Participants were asked to discuss what they perceived as problems with their domestic livestock and their own health. They were also asked about favoured cooking methods to determine if any of those could pose a risk factor. In the case of hunting, hunters were asked to provide information about the seasonality of hunting and the most hunted species to assess the degree of contact and interaction with wildlife. Housewives were asked to describe used cooking methods and how they prepared meat. No data were recorded concerning the people declining to participate in the research.

Semi-structured interviews were used to gather knowledge concerning the above-mentioned topics using open-ended questions. Informants were asked to identify and evaluate several variables under study [15, 26]. Home interviews were conducted by the main investigator. The purpose was to ask people about hunting methods, cooking methods, common diseases for both animals and people, and their personal perception of animal health. For diseases, the morbidity (amount of animals sick with a particular disease) and mortality (amount of animals dead because of a particular disease) were assessed. Interviews were conducted at different times of the day and varied in length according to the situation of those being interviewed. The target population of the study was housewives of varying ages with different backgrounds. Interviewees were asked about their personal opinions concerning different topics. During this stage, direct observations were recorded, if animals were free-roaming within the house, the kind of husbandry they received, if there was an evident clinical disease, the enclosures the animals may have had. Wounds by mechanical means, availability of food or water within house; as well, (if possible), the kind of management people gave to food, how food was prepared, and water storage, among others.

Afterwards, all of this information was categorised according to the topic of interest and compiled in a database (Microsoft Excel) to generate different score matrices. In the case of diseases, clinical signs and clinical findings at necropsy were linked to particular pathologies consistent with the veterinary literature.

The local Management and Conservation Organisation of Uaxactún (OMYC, in Spanish) supported the ongoing investigation with previous information collected by a local census. OMYC regulates the use of forestry resources of the village, organises the activities for collection of goods from the forests through the year, and regulates the market of the products which are generated within the village to other places. Among the data provided by OMYC for this research were data on work activities, salary incomes, house infrastructure, literacy of people, as well as their keeping of animal species, among other information. These data were analysed and the most relevant data are presented.

Various PE techniques were used during interviews. Proportional piling allowed relative scores to be assigned to various categories related to one criterion using maize or beans as designated counters [12]. Proportional piling was used individually rather than in groups because of the low willingness to collaborate of the villagers. For cooking methods, proportional piling was used [15, 26]. A ranking from 3 to 1 was given according to the predominance of the different methods already identified in the community, where 3 was the highest score, indicating a very common method, and 1 was the lowest score, indicating an infrequent method. People could only give two (3 and 1) or the three different scores. In the case of species, scores were added and divided by the number of houses and afterwards relative frequencies were determined for each cooking method [15, 26].

For diseases, respondents were asked to mention diseases present in the village, either by perennial or outbreak status, and the importance they have. Some interviewees were asked to describe the occurrence of diseases as timelines. All data and observations from these PE techniques were collected using written notes. No photographs were taken because of the uneasiness villagers felt towards this. Prevalences for the different diseases in this study are named as “prevalence” for the data obtained by classical epidemiological work [11], whereas for the data estimated by participatory means, “PE-“is the further abbreviation.

Cooking methods were classified with the following categories:

- *Stewing* Combination of solid food ingredients that have been cooked in liquid and served in the resultant sauce. Ingredients in a stew include any combination of vegetables and meat
- *Broth* Liquid food preparation, typically consisting of either water or an already flavoured stock, in which bones, meat, fish, cereal grains, or vegetables have been simmered. Broth is used as a basis for other edible liquids such as soup, gravy, or sauce
- *Grilling* (a la plancha) Is a Latin-American food preparation, consisting of putting the meat directly on a heated iron surface. Usually this is done without oil or fat, leaving meat with a low degree of fat. This is comparable to fried meat, but without adding any additional kind of fat
- *Roasting* Method involving a substantial amount of direct radiant heat, and often used for cooking meat quickly. Food to be roasted is cooked on a grill or griddle. Heat transfer to the food when using a grill is primarily via thermal radiation
- *Frying* Is the cooking of food in oil or another fat. Foods can be fried in a variety of animal or vegetable fats
- *Marinating* Is the process of soaking foods in a seasoned liquid before cooking. The ‘marinade’, can be either acidic (made with ingredients such as vinegar, lemon juice, or wine) or enzymatic (made with ingredients such as pineapple). In addition to these ingredients, a marinade often contains oils, herbs, and spices to further flavour the food items. The process may last seconds or days

Regarding social aspects, and to increase the participation of the community, an active role was played by the field researcher; the veterinarian was working for free at the village providing different services. The most appreciated service to gain integration into the community was deworming and vitaminisation of pigs (albendazole 10 % PO, 20 mg/kg; AD3E+ Complex B 3–5 cc). Other treatments included administering ivermectin (1 %) to dogs, and deworming and vitaminising equines. As well, poultry were vaccinated against Newcastle disease, pasteurellosis, and Coryza disease (Triple Aviar, LAVET laboratories, Guatemala).

For the study, a written request for working in the village was given to the OMYC, where the purpose of the investigation was explained. A letter with the consent of the aforementioned institution was obtained to allow the field work to take place. Afterwards, each participant was informed orally about the purpose of the investigation and information was obtained from individuals who accepted to participate. No written consent was obtained from the individual participants. Work was conducted only with adults. All participants gave consent to use the data due to the anonymity with which it was to be used.

Ethical clearance was not required from the University of Veterinary Medicine Hannover, Germany as only personal opinions were to be collected. Project was discussed and developed with help of, and as well approved afterwards by the Graduate School of the Faculty of Veterinary Medicine, University of San Carlos, Guatemala, and the Zoosanitary Department of the Ministry for Agriculture, Livestock and Food of Guatemala. Both institutions gave a positive review to the project. Afterwards, all permissions to perform the field interviews were sought at and agreed by OMYC after reviewing and discussing the contents of the investigation, as it represents the local community authority and are the responsible for all the affairs concerning to the village.

### Laboratory methods

For the diagnosis of Swine diseases, tests performed were: Microagglutination test for Leptospira (at the University of San Carlos of Guatemala), and ELISA tests for classical swine fever, mycoplasmosis and actinobacillus, as well as card test for Brucellosis, the latter ones being performed at the Ministry for Agriculture, Livestock and Food of Guatemala. For poultry, blood tests were the haemagglutination inhibition test (for Newcastle disease), and immunodiffusion in gel-agar (for avian flu). Samples for poultry were taken under a random by-convenience sampling method, after visiting different houses in the village [11].

### Statistical methods

For the prevalences, the PE-prevalences, morbidity and mortality rates of each disease, mean values were generated from the collected data by means of descriptive statistics. Associations were measured with Cramer’s V and Fisher’s exact tests were used to check for statistical significance of the association. All data were processed in SAS^®^ 9.3 software [24].

## Results and discussion

### General remarks

The informants who agreed to participate represented a subset of the population. Not everyone was willing to participate, and not all respondents gave relevant information. In total, information from 10 hunters and 26 housewives who actively participated in the investigation and gave information was used. Respondents differed in their comfort level when discussing certain subjects. Some respondents ended the conversation several times because of stress or discomfort, whereas some decided not to cooperate before an interview took place.

Hunters were particularly ill at ease during interviews. They explained that they received a lot of pressure from outside the village to stop this activity. Most of them claimed that there were no other jobs for them and the necessity of sustaining a family forced them to go hunting. In contrast, housewives were more open and kind when giving information concerning their cooking methods and experiences with the diseases of their animals.

### Description of community information

Data collected by the OMYC organisation at the village, concerning 82 of 147 families living in the village were analysed.

According to personal communication with the staff of the OMYC, there was no actual difference between being associated with the organization or not, as every villager was entitled to the same benefits independent of this status (i.e. products of the concession, salaries, or employment opportunities). Furthermore, there were several families in which one or more individuals were members of the OMYC, whereas the rest were not. Thus, several families not holding a membership status may be considered as members since an individual was a member.

From the data of OMYC, the association between keeping different animal species is as follows: Poultry and Pigs (Cramer’s V = 0.2080, p = 0.09); Poultry and Horses (Cramer’s V = 0.1543, p = 0.344); Poultry and Mules (Cramer’s V = 0.1055, p = 1.00), Pigs and Horses (Cramer’s V = 0.2266, p = 0.0659); Pigs and Mules (Cramer’s V = 0.0454, p = 0.65) and Horses and Mules (Cramer’s V = 0.2165, p = 0.1102). These results indicate that the ownership of one animal species did not influence the ownership of another one.

### Diseases of domestic animals

Within Table 1, results obtained by Participatory epidemiology (PE), concerning the PE-percentages of morbidity and mortality of each relevant disease named in the village are summarised. According to PE, poultry diseases are the most important as those bear the highest PE-morbidity and PE-mortality rates. For the villagers, both pigs and poultry represent the most important species in the community. Equine diseases appear to be neglected by villagers and problems (apparently strangles) due to *Streptococcus equii* is the only mentioned disease for them. It is important to state that for some diseases, clinical signs and clinical findings at necropsy were linked to particular pathologies consistent with the veterinary literature: Important is to state that these methods are not as accurate as laboratory methods, and may also be considered as biased towards most common diseases reported for the region.

**Table 1:** Participatory epidemiology morbidity and mortality of animal diseases

Disease	PE-morbidity (average) (%)	PE-mortality (average) (%)
Swine dermatitis	4	0.12
Swine parasites	40.80	0.25
Newcastle disease	89	86
Avian pox	38.00	17
Avian diarrhoea	12	4
Equine strangles	7	0.20
Canine mange	23	1
Canine leishmania	10	0.00
Canine botfly (*Dermatobia hominis*)	5.00	0.00

#### Pigs

Pigs are the second most important backyard livestock in the village after poultry. In total, 31.7 % of the households interviewed by the OMYC owned pigs, and 53.6 % considered them as a necessity. Diseases of pigs are not deemed as important as those of poultry since they are not deadly. The most common disease mentioned by villagers was cysticercosis, locally known as “sapillo”. This disease was commonly referred to by villagers as being a major problem. Nevertheless, no evidence of cysticercosis was apparent during field necropsies, and when people were asked if they had ever come across this disease in the community, no one had. Therefore, there is no field evidence of the presence of this disease in Uaxactún. One person commented that the last case had occurred approximately 20 years previously. Governmental organisations have created a high-profile for this disease, and therefore, people are always cautious when consuming pork, checking the meat and carcass. This is an important finding as cysticercosis is present in other communities of the country although the Uaxactún community appears to be free of this disease. Some pigs were dewormed with albendazole and several nematodes were recovered from faeces and later identified as *Ascaris sp*. Those questioned believed that around 40 % of pigs are infested with nematodes, but there is no mortality due to this.

Another condition reported and observed in pigs was dermatitis. A clinical examination of skin was performed to determine if there were any infections or suppurations of skin, if the skin was irritated, sensitive to contact, heat or swollen. Only 3 out of 67 pigs were believed to have skin lesions due to dermatitis. Several others were only covered with dirt which may have been considered as either dermatitis or mange. This contrasts with the results found by Mérida et al. [18], where leptospirosis had a prevalence of 39 %, which could be considered therefore as a subclinical disease.

Since Uaxactún is located 23 km from the Tikal National Park, being approximately 40 km to the closest village—El Caoba—and 85 km from the City of Flores, importation of pigs and other livestock are controlled at the Tikal Security Gate. Pigs brought into Uaxactún usually come from El Caoba. The Peten Region has been free of classical swine fever since 2005 (MAGA, Declared in the Ministerial Agreement 1993–2004), and therefore this disease was not expected to be found. Nevertheless, the surveillance programme had to confirm the absence of this disease. Furthermore, it is forbidden to vaccinate pigs against CSF in the Peten region, and thus ELISA analyses were expected to be negative either to field or vaccination exposure.

#### Poultry

Poultry accounts for the largest domestic livestock within the community, mainly composed of chickens (*Gallus gallus*), Muscovy ducks (*Cairina moschata*), and turkeys (*Meleagris gallopavo*) to a lesser extent, all of various ages. Poultry diseases were considered as the most relevant issue for the community. In total, 85 % of the surveyed families kept backyard poultry, whereas 90 % considered this a necessity.

Diseases mentioned by villagers were Newcastle disease (NCD), locally known as peste or accidente, avian pox (viruela), and various respiratory diseases generally known as soco. Fifty-two blood samples from poultry -33 from chickens, 3 from turkeys and 16 from ducks- were sent to the Reference Laboratory of the Ministry for Agriculture, Livestock and Food (MAGA) of Guatemala; furthermore, 16 swab samples of both trachea and cloaca from ducks were sent to the Laboratory for Ornitopathology, University of San Carlos of Guatemala, for viral isolation.

Following the indication of the National Association for Poultry of Guatemala (ANAVI) and the National Poultry Program (PROSA), attention was given to both NCD and avian influenza (AI), for the national surveillance system. Since there was no vaccination history for sampled poultry, it was assumed that all positive results were due to field exposure. 29 samples from chickens were used for NCD diagnosis and 33 for AI. In total, 12/29 (41.3 %) of chicken samples, 1/3 of turkeys and 1/16 of ducks were positive to NCD. However, none were positive to AI. All swabbed ducks proved negative for viral isolation, and thus NCD virus, AI virus, and infectious bronchitis (*Coronavirus*) were not detected.

For disease identification, villagers reported both depression and respiratory signs as the most common evidence of disease and therefore not relevant for disease discrimination. NCD was linked to high morbidity and mortality rates, in which birds developed respiratory signs, followed immediately in some cases by neurological clinical signs (depression, dropped wings, twisted neck). Almost no poultry survives this disease, and usually, surviving poultry are killed and discarded. Whenever a poultry epidemic occurs people attribute it to NCD, no other diseases are regarded as being as mortal or as important as this one. Avian pox was linked to scabs on the comb, wattles, around the eyes and over the nose. Few cases of Avian Pox were personally seen during field observations, and respondents did not regard it as an important disease unless chicks were affected, as these are highly susceptible to death. Other diseases were considered as unimportant due to a low transmission level or low mortality rates. Diarrohea or respiratory diseases were not defined as a huge problem as only a few chickens in the household would be affected.

A key factor observed regarding disease transmission was that when an outbreak was detected or suspected, people either killed for self-consumption or sold live poultry to their neighbours. Under threat of an outbreak of Newcastle disease, chicken prices decreased by more than 50 %. Therefore, some villagers from other parts of the community found it attractive to buy them, thereby supporting the spread of the disease.

It is important to remark that from August to September 2013, an outbreak of NCD occurred in the village; most of the poultry in the south of the community being found dead. By means of participatory methods, it was determined that poultry from outside the village were taken into a household, and afterwards the disease spread to the surrounding areas. Consequently, a vaccination programme was initiated in collaboration with the OMYC from the other side of the community; the disease did not spread to these areas. Samples of poultry were collected before vaccination.

#### Equine

Horses (*Equus ferus caballus*) and mules (E. f. caballus * E. africanus. asinus) are of low importance in Uaxactún; they are not used for transport purposes or as burden animals. Only 12.2 % of the surveyed population owned horses and 6.1 % mules, Horses and mules were considered as a basic need by 50 and 43.9 % of respondents, respectively. Diseases were primarily by apparent strangles (infection with *Streptococcus equii*), which is usually not treated. High levels of tick infestation were personally observed, but this is usually not treated either. In former times, horses were a means of transport used by xateros and chicleros (forest workers) to move within the forest when roads were not available and access was difficult. In some reported cases, horses were still used to transport various resources, particularly when roads were either flooded or swampy, making cars or other vehicles useless.

#### Dogs

In the case of dogs (*Canis lupus familiaris*), manges (*Sarcoptes sp*. and *Demodex sp*.) were a common problem for which people would regularly ask for medicine (ivermectin) at the veterinary agro-services in the City of Flores. This disease was not considered important by villagers. Colmoyote (human botfly by *Dermatobia hominis*) was described as a problem. Nevertheless, this was not considered relevant by the villagers either. During personal observations, nine dogs were either affected by living larvae or had scars suggesting old infections. Some people claimed to have been affected by this parasite; but only three human cases were personally recorded by the researcher.

An important disease diagnosed at clinical level on 13 dogs was leishmaniasis. This disease appeared to affect dogs frequently and humans to a lesser extent as no human cases were personally seen during the fieldwork. However, a few people claimed to have had leishmaniasis in the past.

#### Miscellaneous

By law, holding any kind of ruminant is forbidden for community forestry concessions of the Maya Reserve Biosphere. Furthermore, some pigs and equine are frequently injured with a machete or other stabbing weapons by villagers when animals invade the crops of other people; one horse and three pigs were injured during this study period.

#### Seasonality of diseases

Seasons were divided into the dry (summer) and rainy (winter) season. The dry season usually extends from mid-March to the end of May, whereas the rainy season extends from August to January, with a varying intensity during this time. The other months show progressively changing weather and are considered neither as summer nor winter. It is worth mentioning that for 2014, people claimed seasonal variations to be uncommon as there were several storms close to the region during that year.

The summer season was mentioned as a time when outbreaks of poultry diseases such as NCD and avian pox occur. Various outbreaks may happen throughout the year, but mostly during this season. No steps to prevent these diseases were taken by the community.

As previously mentioned, during August there was an uncommon outbreak of NCD, involving fieldwork for the researcher. The vaccination programme conducted during those days may have suppressed the occurrence of Newcastle disease during the 2014 summer season.

### Hunting

As stated by Baur in 2008, hunting was a common activity happening on an irregular basis and focused on large vertebrates without any concern for the sex or species. According to the information generated by OMYC, hunting accounted for approximately 0.75 % of the income, founded on the perception of the surveyed families, and thus represented a low proportion of the local economy according to villagers. This variation in estimates may rely on the fact that hunters did not feel at ease while talking about this topic; additionally, since there is no sudden income due to this activity, they would not be able to measure the amount that hunting represents. Hunters could not account for an average number of animals per month or season as this number varied depending on several factors including time spent in crop fields, other activities, health issues or family affairs.

As a general background, nine out of the ten hunters had backyard animals, of which only one had vaccinated some poultry against NCD. One hunter had a child who hunted as well, and five of them would like to receive support with the health or management of their animals. Of the hunters, six stated that they would visit the clinical centre when clinical disease occurred; four claimed they did not require the help of a practitioner. Seven had experienced diseases during the previous year. Common diseases mentioned by hunters affecting them were respiratory symptoms, diarrohea and flu. Usually, no further diagnoses were performed at the village but treatment was focused on clinical symptoms.

### Hunting seasons

Hunting was influenced by season; the summer season was described as the best season for hunting peccaries, as most of these herds would gather close to water sources which do not dry up during this season, and which were known by the hunters. White-lipped peccaries (*Tayassu pecari*) were regarded as the easiest species to hunt during summer months due to the high number of individuals composing a herd. During the rainy season, hunting of peccaries happened by chance in an opportunistic pattern as they roamed freely in the forest due to water and food resources being readily available everywhere.

In contrast, deer (*Mazama sp*., *Odocoileus virginianus*) were present all through the year, being elusive animals which usually were harder to hunt during the dry season, but easier to do so during the rainy season as there are no dry leaves or branches on the soil creating noise, allowing for a silent approach to the individuals, which usually live alone; access to the forest, however, being more difficult.

Armadillos (*Dasypus novemcintus*) and Tepezcuintles (*Cuniculus paca*) were hunted whenever they were found, usually not by purposive hunting but by chance, when tracks were spotted and hunters would return on another trip. Tepezcuintles were sometimes hunted with the aid of dogs, whereas armadillos were not. Central American agouti (*Dasyprocta punctata*) was regarded as an undesirable species to hunt.

The rainy season as well allowed an easier approach to birds, which may be over the soil or on the branches of trees. In the case of birds, the curassow (*Crax rubra*) and crested guan (*Penelope purpurascens*) were the most hunted species as those are the most abundant. Hunters prefer hunting curassows because of their taste and size (3–5 vs 1.6–2.5 kg; [23]). There is a high degree of sexual dimorphism of curassows and hunters usually prefer to hunt males, claiming that if females are hunted, no offspring would be available for the following years.

The people did not consume any reptiles (Reptilia: Vertebrata) and only a few fishes inhabiting the nearby rivers or lakes.

Of the surveilled hunters, five had dogs which were regularly used to hunt under particular circumstances (i.e. hunting for a short time, elusive animals, with a knowledge of prey at a precise moment and location); the use of dogs to assist hunting was never consistently successful, even if all hunters claim that dogs increase the success of hunting. As well, hunters said that bringing along dogs may be dangerous as jaguars (*Panthera onca*) or pumas (*Puma concolor*) could attack. Likewise, if peccaries feel distressed, they may attack the dog and the hunter. Hunters stated that the greatest risk when hunting were the pit-viper species (Crotalinae: Reptilia), particularly Fer-de-Lance (*Bothrops asper*), rattlesnakes (*Crotalus simus*), and jumping vipers (*Atropoides mexicanus*).

#### Hunting patterns

Among the ten hunters interviewed, two who hunted 1–2 times a month, one hunted 3–5 times a month, two hunted 1–2 times a week and four hunted 3–5 times a week. One hunter said he hunted less than once a month. Three hunters stated that they prefered hunting together, whereas four hunted with friends or relatives, two only with friends, and only one went either alone or accompanied. Transportation means were diverse as most preferred going into the forest on foot or by vehicle (motorcycle, bicycle) if available.

Two hunters stated that they only hunted for self-consumption, whereas eight claimed hunting for both self-consumption and selling purposes. Most hunters sold only to relatives and close friends. Concerning the frequency of hunting, five hunters stated that they did not hunt more because of their responsibilities, four because of the low availability of animals, and one claimed to hunt what was needed. Five hunters brought along dogs to hunts, depending on the weather conditions and former experiences of previous days.

Depending on the kind of animal hunted, the carcasses was either prepared in the field (large mammals) or the animals were brought complete to the village (tepezcuintle, armadillos, wild birds), the latter only being prepared on the field if the hunter was returning to the village on the following day. If a carcass was brought to the village on the following day, it would be roasted on site for conservation.

As stated by Alvard et al. [1]; hunting is an important component of native subsistence strategies. It may be found from the Americas to Africa as a widespread phenomenon in tropical forests [22]. Subsistence hunting varies greatly from one region to another depending on factors such as habitat, land use, current status of wildlife and cultural factors. Although subsistence hunting is ubiquitous in Latin America, information on it is scarce and mostly anecdotal. The most specific studies only cover the forest areas of Peru and Brazil [21].

In most cases, hunting pressure is higher than the sustainable harvest amount [1, 21, 22]. Peres [22] describes for 24 locations in both the Brazilian Amazon and regions of Perú the pressure of hunting, and considers through a socioeconomic approach the enormous value that game meat represents for those regions. These statements may also apply for the forestry of Uaxactún, even if forests and communities are different, the economic problems that these villages may share may be similar, and could lead to the same forestry approach.

### Cooking

Figures for cooking methods are presented in Table 2. All figures are estimated percentages, by species, of what each cooking method would represent for meat cooking. In general, people did not eat raw meat or medium-rare meat, nor if it is still bloody. Scores are added by species and households and relative frequencies for each cooking method are presented.

**Table 2:** Scoring results (percentages) of cooking methods at Uaxactún

	Stewing	Broth	Grilling	Roasting	Frying	Marinating
Poultry	42	13	6	18	21	0.00
Beef	8	36	35	18	3	0.00
Pork	9	0.00	19	22	5	45
Wild birds	51	23	8	18	0.00	0.00
Peccaries	53	16	0.00	31	0.00	0.00
Deer	31	2	44	21	2	0.00
Tepezcuintle	71	0.00	1	0.00	28	0.00
Armadillos	46	0.00	0.00	54	0.00	0.00

As shown in Table 2, for the case of poultry, wild birds, pork, peccary and tepezcuintle, more than 50 % of cooking methods reach high temperatures (stewing and broth), which could be considered as a protective factor against food-borne pathogens. Methods such as grilling may be considered protective since meat is heated to high temperatures on an iron and according to the duration of cooking there would be no raw or medium-rare meat. Roasting, conversely, could represent a problem if direct exposure to the flames burnt the external layer of food, prompting cessation of cooking, but leaving raw parts or tissue liquids; armadillos were usually roasted and may represent an exposure of this kind. Nevertheless, as corroborated during fieldwork, people did not consume meat until completely cooked. Surveyed people said that roasting took place over a low fire (when no flames came from the logs, but only heat).

As there was no electricity in Uaxactún, food conservation is not possible using fridges or under conventional means. Therefore, meat (of domestic or wild origin) was either smoked or grilled, then being dried and stored in pots covered with foil. On some occasions, large iceboxes were filled with ice for storage for a maximum of 2 or 3 days. Other preparations were possible, but only lasted for 1 day. At the first indication of spoilage, meat was discarded.

For the viscera of hunted animals, liver was the most commonly consumed viscera, followed by lungs, locally named as “bofe”. The remaining viscera were discarded because they were not considered edible. Some people may eat kidneys. However, kidneys were usually not eaten, and when they were, were well cooked, so that leptospira was not likely to survive the cooking procedure. Intestines of domestic swine were used to prepare sausages, but not the intestines of wild animals as they are usually infested with worms.

Lungs of peccaries were said to always have worms. In that regard, 10/11 in-depth inspected peccaries were positive for lung worms, possibly *Metastrongylus apri* (based on gross examination by size and morphology) [25]. Lungs of other wild animals as well as of domestic swine are not infested with any worms. No liver was positive for parasites and no reports of this were given.

Concerning to the meat consumption in the village, Table 3 gives an estimate of the times that meat is consumed by the villagers, per week. Furthermore, concerning the proportion of bush meat within the diet, 43 % of housewives indicated that most of the meat consumed at home was bush meat (Table 4). These data, nevertheless, might be considered as biased, as most of the consulted housewives were relatives of hunters. Hunters would usually share the carcass of the hunted animals with their closest relatives, this being the case for this subset of the population, and may not be completely representative for the village.

**Table 3:** Meat consumption per week

	n	Percentage
1 time a week	1	4
2–3 times a week	8	31
4–5 times a week	13	50
6–7 times a week	4	15
Total	26	100

**Table 4:** Proportion of bush meat within the diet

	n	Percentage
Missing	1	4
Rare (1–20)	4	15
Some (21–40)	4	15
A fair amount (41–60)	5	19
A lot (61–80)	1	4
A great amount (81–100)	11	43
Total	26	100

In the case of water, people would frequently consume rainwater, which they collected during the rainy season in large storage containers. Water that people usually collected from nearby ponds was used for showering or cleaning. When required, most people used pondwater after a sediment had deposited without any further steps for purification, whereas some other people either added drops of chlorine or boiled the water before consumption. Table water is virtually absentf in the community and almost no one obtains bottled water.

Regarding fruits and vegetables, people usually washed them before consumption. As well, cooking utensils would be disinfected by means of soap before and after using them for cooking.

Kuhnlein et al. [14] report in depth studies of the food systems of indigenous peoples for several regions of the world, as well as the relevance they have as potential protective factors during rural approaches, as well as the fruitful impact of simple capacitation over health and hygiene recommendations. Warnock [27] reported some cooking methods in Cambodia for the World Health Organization. He assessed the degree of hygienic practices, if meat was cooked thoroughly, if raw and cooked food were separated during preparation, and if people washed their hands. He describes how the education of people through capacitation may change perception of the importance of proper cooking and handling, as well as how hygienic practices may increase as long as they do not mean any further costs for the villagers.

Kuhnlein et al. [14] describe as well the participatory approach used in nine different scenarios in Canada, India, Peru, Japan, Nigeria, Kenya, among other countries, where the health assessment was driven by an approach of understanding cooking and where health improvements were made based on this assessment. There is an important description concerning the epidemiological approaches in these studies, not only for understanding the cultural and demographic characteristics of the people and the nutritional basis given by their cooking styles, but as well as for teaching them a better management for raising their animals, and the latter measure of the impact of social activities for improving health and management of their livestock, as well as for the hygienic practices concerning food preparation.

### Medical and veterinary services

Within Uaxactún there was a clinic (human health centre) which most villagers visited in case of disease. Nevertheless, there were no reliable databases containing information on the cases diagnosed at the clinic. Common infectious problems affecting villagers were diarrohea and respiratory diseases. Most treatment was directed towards controlling clinical symptoms instead of defining a specific diagnosis. This was partially due to individuals failing to seek medical attention because severity of diseases was low, or embarrassment of visiting the medical staff as well as the lack of equipment and facilities for further diagnoses. Pathogens such as Salmonella, *E. coli*, Campylobacter, Giardia, among other Enterobacterias and parasites, might be partially responsible for provoking these symptoms. Nevertheless, there was no database for corroborating both the incidence and the distribution of the pathogens frequencies.

Samples for tuberculosis were commonly taken although no positive cases were reported. As well, blood samples were collected when “oreja de chiclero” (leishmaniasis) was suspected.

Concerning veterinary services, the villagers of Uaxactún expressed little interest in having such services. Villagers were concerned about the health of their animals; but concurrently, they were not willing to travel far or pay for those services. Some villagers purchased NCD vaccination (ocular drops). This was a remarkable finding as few people are willing to invest in the health of their backyard animals. People invested in poultry but not in other animals due to the high morbidity and mortality rates of NCD. There were just a few non-governmental organisations which help with vaccination, particularly poultry. Programmes for de-worming and a multi vitamin-injection programme for pigs provided by the researcher in appreciation for being allowed to work in the community were well received. People noticed that after treatment pigs had a larger appetite and were more active; this was attributed to malnourishment and heavy infestation of digestive nematodes.

Fay [13] has a wide approach to the health of poor people in Latin America; she remarks how in Guatemala the urban poor have a better health performance than their rural counterparts, whereas in other countries it happens to be the opposite. She addresses how the physical environment, along with inadequate hygiene practices deters health. It is mentioned, nevertheless, that the individuals’ health-seeking behaviour is a relevant factor for the health improvement. She presents data revealing the relationship between illnesses on the one hand and income, unemployment, education, and access to basic services on the other. The data neither prove causality nor isolate the influence of each variable, but they do demonstrate a positive correlation between health and factors associated with poverty.

In addition, although the evidence is not specific to urban areas, health care systems in Latin America provide an unequal distribution of benefits: The gap between the need for and utilisation of services is much larger among the poor. Increasing access to health care services may not increase the utilisation of them since the poor do not take advantage of health services to the same extent as the rich. (Suarez-Berenguela 2000, in Fay [13]) The former statement applies to the situation in Uaxactún. Even if medical services and medicine are available for free in the village, people fail to seek medical advice. These results suggest that education programmes must accompany increased access to services in order to maximise their benefits. Health is as well linked to food management and hygiene [14]; and therefore an improvement in health may be achieved by educating the people concerning disease transmission through food.

### Summary discussion

The present study generated information concerning wildlife diseases and factors influencing hunting, as well as the particular seasonality of diseases of livestock within the area of Uaxactún, Maya Reserve Biosphere, Guatemala. However, due to the limited resources, as well as being the first study of this type in the region, it should be considered as a pilot project which may be improved and extended.

Many times during the fieldwork development, some information was difficult to analyse or synthesise. Several biases may be expected from the fieldwork, as most of the information corresponds to the opinions of (in most of the cases) illiterate or people with a minimum amount of education. As well, for some cases, there was a lack of interest of people in giving information, or information was changed as the interview progressed. Added to the lack of interest, mistrust was as well a key topic which hindered the degree of openness for the information given, but as well, the veracity of the information may have been lowered by mistrust. To improve this, interviews occurred in a personal and private environment, where trust and openness was enhanced; nevertheless, in many cases (as identified later), several people felt ashamed and expressed several opinions in many cases as they thought their opinions would be considered as pointless. It is likely that in group activity results may differ from results of individually conducted interviews, but it is not possible to contrast the impact of social desirability bias in the case of group procedures to the restricted knowledge bias in the case of the individual procedures used in this study. Involving people to describe their problems and propose solutions would enhance the success of an investigation.

Additionally, there is a degree of bias for the diseases diagnosed in the village as for the case of most species; diseases were diagnosed at clinical level, with no further effort made to try to isolate any pathogen or carry out any further research. In the case of the participatory epidemiology, an effort to corroborate the information of the diseases was made as the fieldwork took place.

Regarding animal diseases within this study, we conclude that there are no immediate threats to public health from the domestic livestock and that the level of problems is not substantial as to require immediate action. The most representative diseases for livestock are Newcastle disease and avian pox, for which control and prevention would be possible and feasible if the necessary steps for the regular vaccination of poultry are programmed between the community and the government -or with a non-governmental organisation. Other procedures, such as deworming or vitaminisation of domestic livestock might be conducted by villagers themselves after proper capacitation is given. Further steps are desirable for a wider study of backyard animals to be carried out, increasing the collection and variety of samples of the different animal species within the village.

Concerning human diseases, no relevant diseases were mentioned among villagers other than respiratory symptoms or diarrohea. There are no definitive diagnostics for these problems and only clinical symptoms are treated. Disease surveillance in humans is required to reveal factors influencing the appearance and frequency of diseases in the region.

We conclude as well that villagers realise the importance of proper cooking, even if this is not recognised by them as being directly linked to diseases. Describing cooking methods indicated several protective and risk factors within the procedure, which may be considered for further health suggestions. Thus, even if roasted and grilled meat might pose a higher degree of risk of raw meat remaining, people took care to cook meat properly before consumption, which inactivated most of the possible pathogens which could be transmitted by meat.

Xate collection (economically important understory palms within the regional forests, of the species *Chamaedora elegans*, *C. oblongata*, *C. erumpents*; Belize botanic gardens [5]) and agricultural activities may influence hunting as these activities put people in direct contact with wild animals. Therefore, understanding hunting as a whole from the reasons generating this activity to the field activities is a necessary step for improving wildlife management and conservation. Increasing the number of other activities and generating other sources of income in the village would decrease the frequency of hunting, thus decreasing the frequency of exposure to the risks related to this. We conclude, too that Xate collection, OMYC and Governmental work as well as agriculture are the most important income activities for the villagers.

As participatory epidemiology relies heavily on indigenous knowledge and terminology, it is recommended that participatory rural appraisal techniques are actively integrated into the surveillance systems in rural communities. Within participatory epidemiology, the main methods for collecting information are by semi-structured interviews, scoring and ranking, and visualisation [26]. Thrusfield mentions how PE has been widely used for data collection and further veterinary actions in several countries in Africa, procedures which have helped either to describe [8, 17, 19], detect [16], control or eradicate [6] several animal diseases, as well as to improve animal health [20].

### Future steps

Regarding the management of livestock for both health and reproduction as well as for improving animal husbandry educating villagers is required. Education concerning food-borne diseases is desirable, as this is an important factor for the prevention of the spread of diseases. To this, capacitation of villagers to apply different drugs and medication for animals as preventive steps may be added as well. Furthermore, education of people for collaboration in this type of studies is desirable as mistrust hinders the development of participatory approaches.

For assessing human and animal health, as well as for analysing the frequency and occurrence of human and animal diseases *one*-*health programs* would be an important step. Increasing the capacity for sample collection and conservation, laboratory diagnostics, and socio-economic analyses may be added to strengthen the spectrum of such a programme. As well, increasing the monitoring and surveillance efforts for different diseases by a participatory approach, allowing people to feel included in the development of health programmes for their communities, may increase the success rate of the fieldwork. Acquiring qualitative information of this kind would be helpful for complementing other information of a quantitative nature. The assessment of particular diseases affecting backyard livestock and human health is paramount for developing health and management plans according to the needs of each particular place.

Besides this, a standardised collection, classification and *storage of information* concerning the different reports of diseases, as well as other data related to the epidemiological tracing and tracking records for a particular outbreak or case is desirable. This should generate a one-health data structure as the basis for action plans and surveillance.

## Abbreviations

AI: avian influenza
ANAVI: sp. National Association of Poultrymen of Guatemala
MAGA: Ministry for Agriculture, Livestock and Food, Guatemala
MBR: Maya Biosphere Reserve
NCD: newcastle disease
OMYC: sp. Management and Conservation Organisation of Uaxactún
PE: participatory epidemiology
PROSA: National Poultry Programme
